# Transcriptome-metabolome analysis reveals how sires affect meat quality in hybrid sheep populations

**DOI:** 10.3389/fnut.2022.967985

**Published:** 2022-08-11

**Authors:** Bowen Chen, Yaojing Yue, Jianye Li, Jianbin Liu, Chao Yuan, Tingting Guo, Dan Zhang, Bohui Yang, Zengkui Lu

**Affiliations:** ^1^Key Laboratory of Animal Genetics and Breeding on the Tibetan Plateau, Ministry of Agriculture and Rural Affairs, Lanzhou Institute of Husbandry and Pharmaceutical Sciences, Chinese Academy of Agricultural Sciences, Lanzhou, China; ^2^Sheep Breeding Engineering Technology Research Center of Chinese Academy of Agricultural Sciences, Lanzhou, China

**Keywords:** sheep, hybridization, transcriptomics, metabolomics, meat quality

## Abstract

Crossbreeding improves and enhances meat quality and is widely used in sheep production; however, the molecular mechanisms underlying the meat quality of various crossbred sheep remain unknown. In this study, male Southdown, Suffolk and Hu sheep were crossbred with female Hu sheep, and the transcriptomes and metabolomes of the longissimus dorsi muscle of the F1 generation were sequenced to explore how different sire breeds affect meat quality. The results showed that 631 differentially expressed genes and 119 significantly altered metabolites contributed to muscle development characteristics and meat quality-related diversity (*P* < 0.05). These genes and metabolites were significantly enriched in lipid metabolism pathways, including arachidonic acid metabolism and PPAR signaling. Several candidate genes were associated with muscle growth, such as *MYLK3*, *MYL10*, *FIGN*, *MYH8*, *MYOM3*, *LMCD1*, and *FLRT1*. Among these, *MYH8* and *MYL10* participated in regulating muscle growth and development and were correlated with meat quality-related fatty acid levels (|r| > 0.5 and *p* < 0.05). We selected mRNA from four of these genes to verify the accuracy of the sequencing data *via* qRT-PCR. Our findings provide further insight into the key genes and metabolites involved in muscle growth and meat quality in hybrid sheep populations.

## Introduction

As consumers increasingly focus on how nutrition, safety, and high-quality meat products affect health, demand for high-quality sheep meat is growing in most areas of China. Studies have shown that crossbreeding can improve and enhance meat quality. Crossbreeding between Duroc and Polish Landrace sheep can improve tenderness (1), and hybrid Berkshire × QingYu pigs exhibited improved carcass quality, with a significant increase in lean percentage and reduced sebum percentage (2). Sang et al. showed that heterosis from crossbreeding can yield high meat quality (3). Southdown and Suffolk are specialized for meat production and exhibit early growth and excellent meat quality; these sheep are recognized as the most popular breeds for meat production (4, 5). Hybridization experiments revealed that introducing high-quality Suffolk and Southdown males for breeding greatly improved their meat production performances (4–6). Therefore, excellent sire hybrids can improve meat quality and meat production performance to meet consumer demand.

Multi-omics association analysis is being increasingly used in complex trait analyses to explore the underlying molecular mechanisms of sheep crossbreeding (7, 8). Using transcriptomic data to obtain many differentially expressed genes (DEGs) and performing association analyses of different metabolites in metabolomic studies enables identifying key gene targets, metabolites and metabolic pathways. Subsequently, a core control network can be built to comprehensively analyze complex mechanisms involved in developmental biology. Transcriptomic and metabolomic analyses are powerful tools for studying meat quality traits and contribute to better understanding the mechanisms regulating muscle growth and development and fatty acid metabolism (9, 10). Multi-omics techniques are widely used to analyze muscle tissue in sheep, pigs and cattle and have identified many genes involved in fatty acid metabolism (11–14). RNA-seq and qRT-PCR have identified several candidate genes for lipid metabolism, such as *ACLY*, *ADIPOQ*, *ELOVL6*, *LEP* and *ME1*, as main gene factors defining the processes influencing meat composition and quality (15).

Hu sheep, a unique local sheep germplasm resource in China, which is known for its excellent characteristics, including perennial estrus, high fecundity, good lactation performance and strong adaptability, is an important dam breed in commercial hybrid mutton sheep (16). Suffolk and Southdown are famous sheep breeds with perfect meat production performance and usually used as sire breeds in hybrid mutton sheep. To reveal how different sire breeds affect meat quality among various crossbred sheep populations, we bred native female Hu sheep with male Hu, Suffolk and Southdown to hybridize these sheep. We then conducted a comprehensive analysis of the transcriptomics and performed two untargeted metabolomics studies on the longissimus dorsi muscles of the first-generation hybrid sheep to provide a theoretical basis for maintaining and improving mutton quality and yielding high-quality hybrid offspring.

## Materials and methods

### Animals and sample collection

We used three groups with six healthy sheep per group to yield the following cross combinations: Southdown × Hu (NH), Suffolk × Hu (SH), and Hu × Hu (HH), those animals were obtained from Gansu Qinghuan Sheep Breeder Co., Ltd., (Qingyang, Gansu, China). We used 18 rams (1 year old) in this experiment, and all rams received the same feeding management and dietary levels. Sheep weights were similar among the groups (HH: 52.83 ± 4.06 kg, NH: 58.06 ± 3.58 kg, SH: 65.51 ± 4.64 kg). The animals were slaughtered after 24 h with food deprived and had free access to water. Subsequently, the longissimus dorsi between the 12 and 13th rib was excised from all 18 sheep on the left side of each carcass within 30 min after exsanguination. Any visible external fat and connective tissues were trimmed before the following analyses.

### RNA-seq data analysis

Muscle tissues from the sheep were used to extract the total RNA using a TRIzol reagent kit (Invitrogen, Carlsbad, CA, United States) per the manufacturer’s protocol. RNA quality was assessed on an Agilent 2100 Bioanalyzer (Agilent Technologies, Palo Alto, CA, United States) and checked using RNase-free agarose gel electrophoresis. After extracting the total RNA, eukaryotic mRNA was enriched with Oligo (dT) beads, and prokaryotic mRNA was enriched by removing the rRNA using a Ribo-Zero™ magnetic kit (Epicenter, Madison, WI, United States). The enriched mRNA was fragmented into short fragments using fragmentation buffer and reverse transcribed into cDNA with random primers. Second-strand cDNA was synthesized using DNA polymerase I, RNase H, dNTP and buffer. The cDNA fragments were purified using a QiaQuick PCR extraction kit (Qiagen, Venlo, Netherlands), end-repaired, A-base-added, and ligated to Illumina sequencing adapters. The ligation products were size selected *via* agarose gel electrophoresis, PCR amplified, and sequenced by Gene Denovo Biotechnology Co., (Guangzhou, China) using an Illumina NovaSeq6000. The raw reads were filtered, and the clean reads were mapped to the reference sequences using HISAT2.2.4 software (17). Gene expression levels were calculated using the FPKM method (fragment per kilobase of transcript per million mapped reads). Finally, differential expression analysis between two groups of RNA was performed using DESeq2 software (18). Genes/transcripts with *P* < 0.05 and |log-fold change| ≥ 2 was considered differentially expressed genes (DEGs). Gene Ontology (GO) and Kyoto Encyclopedia of Genes and Genomes (KEGG) pathway enrichment analyses of the DEGs were performed using R based on hypergeometric distribution. GO terms and KEGG pathways with *P* < 0.05 were considered significantly enriched.

### Metabolite extraction for LC-MS/MS analysis

To extract the metabolites, the freeze-dried samples were dissolved in methanol and concentrated to dry in a vacuum after centrifugation. The samples were then dissolved with 80% 2-chlorobenzalanine and filtered [20 μL of each sample was used for quality control (QC)]. Chromatographic separation was performed in a Thermo Ultimate 3000 system equipped with an ACQUITY UPLC^®^ HSS T3 (150 × 2.1 mm^2^, 1.8 μm, Waters) column maintained at 40°C. The autosampler temperature was 8°C. The analytes were gradient eluted with 0.1% formic acid in water (C) and 0.1% formic acid (D) in acetonitrile or 5 mM ammonium formate in water (A) and acetonitrile (B) at a flow rate of 0.25 mL/min. After equilibration, 2 μL of each sample was injected, and the following increasing linear gradient of solvent B (v/v) was used: 0–1 min, 2% B/D; 1–9 min, 2%–50% B/D; 9–12 min, 50%–98% B/D; 12–13.5 min, 98% B/D; 13.5–14 min, 98%–2% B/D; and 14–20 min, 2% D + the positive control. The ESI-MSn experiments were performed on the Thermo Q Exactive mass spectrometer with spray voltages of 3.8 and −2.5 kV in positive and negative modes (14∼17 min, 2% B-negative model), respectively. Sheath and auxiliary gases were set at 30 and 10 arbitrary units, respectively. The capillary temperature was 325°C. The analyzer scanned over a mass range of 81–1,000 m/z for a full scan at a mass resolution of 70,000. Data-dependent acquisition (DDA) MS/MS experiments were performed with an HCD scan. The normalized collision energy was 30 eV.

### Metabolite extraction for GC-MS analysis

To extract the metabolites, 50 mg (± 5%) of the samples were weighed and transferred into 2-mL Eppendorf tubes. Next, 0.5 mL of an acetonitrile: isopropanol: water (3:3:2, v/v/v) mixed solution (−20°C) and 3–42-mm zirconium beads were added and placed in a high-flux tissue grinder, shocked at 30 Hz for 20 s, allowed to stand for 10 s, cycled eight times, and sonicated in an ice water bath for 5 min. Another 0.5 mL acetonitrile: isopropanol: water (3:3:2, V/V/V) solution was added on an ice water bath, sonicated for 5 min, and centrifuged at 12,000 rpm for 2 min. Next, 500 μL of supernatant solution was added to a new 2-mL Eppendorf tube, the vacuum concentrator was concentrated to dryness (8–10 h), and the remaining supernatant was placed in a −80°C freezer for backup. Next, 80 μL of 20 mg/mL MEOX solution was added for redissolution, vortexed for 30 s, and incubated at 60°C for 60 min. Finally, 100 μL BSTFA-TMCS (99:1) reagent was added, then reacted at 70°C for 90 min and centrifuged at 14,000 rpm for 3 min, then 90–100 μL of supernatant was added to the detection bottle. Samples were placed in sealed cuvettes to be tested and processed for GC-TOF upper detection within 24 h.

Gas chromatography was performed on a DB-5MS capillary column (30 m × 250 μm i.d., 0.25 μm film thickness, Agilent J and W Scientific, Folsom, CA, United States) to separate the derivatives at a constant flow of 1 mL/min helium. Next, 1 μL of sample was injected in split mode at a 1:10 split ratio by the autosampler. The injection temperature was 280°C, and the transfer-line ion source temperatures were 320°C and 230°C. From an initial temperature of 50°C, the temperature was increased at 15°C/min to 320°C for 0.5 min and then held at 320°C for 9 min. Mass spectrometry was performed using a full scan method with a scan rate of 10 spec/s, electron energy of −70 V, and a solvent delay of 3 min (19, 20).

### Statistical analysis of the metabolites

Metabolites were distinguished by comparing the m/z values of the precursor ions, retention time, and fragmentation patterns with the standards in a database compiled by Gene *Denovo* Biotechnology Co., Ltd., Metabolic alterations among the groups were analyzed *via* principal component analysis (PCA) and (orthogonal) partial least-squares discriminant analysis (OPLS-DA) after data preprocessing by mean centering (Ctr) and Pareto variance (Par) scaling, respectively. Metabolites were identified with *P* < 0.05 by *t*-test, and metabolites with a VIP ≥ 1 were considered differential between the groups. Identified differential metabolites were subjected to metabolic pathway analysis through the KEGG database.

### Joint analysis of transcriptomic and metabolomic data

Pearson correlation coefficients were calculated between the SCMs and DEGs *via* pairwise comparison using the Hmisc package in R^1^. DEGs and SCMs with a threshold of |r| > 0.5 and *P* < 0.05 were considered significantly correlated and were subjected to conjoint biological annotation using the KEGG database. Here, only the pathways containing DEGs and their highly related metabolites were selected. The networks were visualized using Cytoscape^2^.

### Real-time quantitative PCR

qRT-PCR was performed per the manufacturer’s protocol (TransStar Tip Green Qpcr SuperMix, Transgen, cat. no. AQ141). The reactions were performed in 20 μL volumes on a Bio-Rad C1000 Thermal Cycler. The qRT-PCR procedure was 94°C for 30 s, 40 cycles of 94°C for 5 s, 60°C for 15 s and 72°C for 10 s. β-actin was used as a reference gene to normalize gene expression. Supplementary Table 1 lists the primers used for the qRT-PCR. Fold-change was calculated for each candidate gene, and the sample was calculated using the 2^–ΔΔ*Ct*^ method. Statistical analyses were performed using GraphPad Prism 8. Quantitative data are presented as the mean ± standard error of the mean. The significance level was calculated using one-way analysis of variance and Tukey’s *post hoc* test. *P* < 0.05 was considered statistically significant.

## Results

### Identification of differentially expressed genes and functional enrichment analysis

**FIGURE 1:**
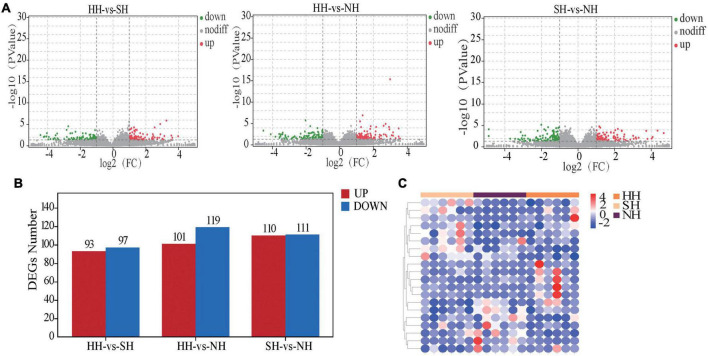
Transcriptomic comparisons of the longissimus dorsi for the HH, SH and NH sheep. **(A)** Volcano plots of the DEGs, non-diff: non-differentially expressed genes. **(B)** Statistical map of the DEGs. **(C)** Cluster heatmap of the DEGs, red: upregulated genes; blue: downregulated genes.

To better understand how different sire breeds affect meat quality among three hybrid sheep populations, we analyzed the muscle transcriptomes using comparative RNA-seq. After high-throughput sequencing and filtering the raw reads for quality control, we obtained 28.53, 26.10 and 27.46 million high-quality clean reads for the HH, NH and SH sheep, respectively (Supplementary Table 2). For each sample, > 91% of the reads could be mapped to the reference sheep genome (Oar. v4.0^3^). We obtained 631 DEGs in total for the HH vs SH, HH vs NH, and SH vs NH comparisons (Supplementary Table 3), the results of DEGs were presented in a volcano plot (Figure 1A). The HH vs SH comparison yielded 190 DEGs, including 93 upregulated and 97 downregulated genes; the HH vs NH comparison yielded 220 DEGs, including 101 upregulated and 119 downregulated genes, and the SH vs NH comparison yielded 221 DEGS, including 110 upregulated and 111 downregulated genes (Figures 1A,B). A heatmap of the DEGs showed that the samples were well clustered (Figure 1C). After clustering, the genes were mainly divided into three groups and were differentially expressed among the three comparative groups.

**FIGURE 2:**
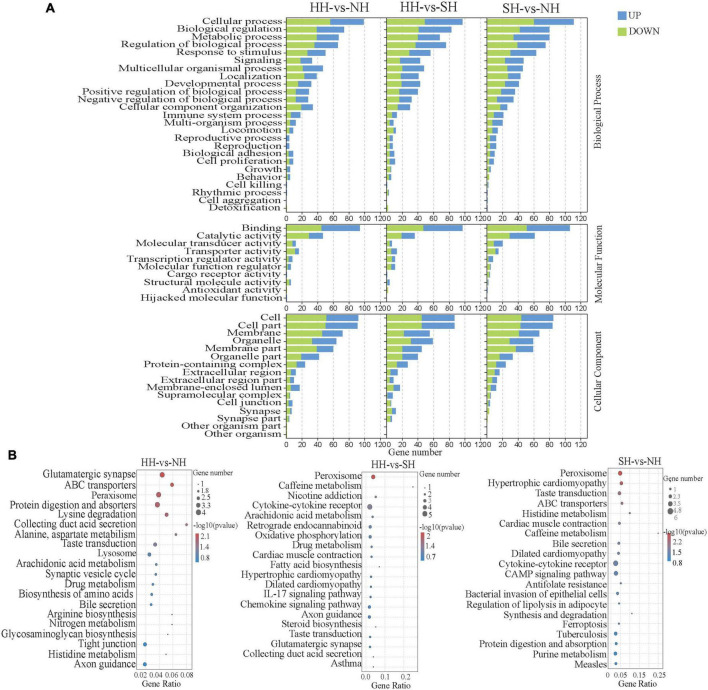
Functional enrichment analysis of the longissimus dorsi samples of the HH, SH an NH sheep. **(A)** GO analysis of the DEGs in the three groups. The ordinate indicates the GO terms. **(B)** Bubble diagram of the top 20 KEGG pathway enrichments for the HH-NH, HH-SH, and SH-NH comparisons. The ordinate indicates the pathways.

To further understand the functions of the DEGs, we performed functional enrichment analysis (Figure 2A), which yielded 51 enriched GO terms, including 25 biological process (BP) terms, 10 molecular function (MF) terms and 16 cellular component terms (CC). The enriched terms were potentially related to growth, development, and meat quality, including multicellular organismal process, metabolic processes, developmental processes, cell proliferation and growth (biological processes); antioxidant activity, molecule function regulator, and catalytic activity (molecular functions); and cell parts and organelles (cellular component), including some genes already reported in the literature that were also detected in our RNA-seq analysis, such as *MYLK3* (myosin light chain kinase 3), *MYL10* (myosin light chain kinase 10), *FIGN (Fidgetin)*, *MYH8* (neonatal myosin heavy chain 8), *MYOM3* (Myomesin-3), *LMCD1* (Homo sapiens LIM and cysteine-rich domains 1), *FLRT1* (Fibronectin leucine rich transmembrane) and other genes (Supplementary Table 4). Figure 2B shows the top 20 significantly enriched pathways among the DEGs (*P* < 0.05). KEGG enrichment results showed that the DEGs for all three groups were mainly enriched in pathways related to fatty acid metabolism, including arachidonic acid metabolism, fatty acid biosynthesis and regulation of lipolysis in adipocytes.

### Metabolomic profiling based on LC-MS/MS

**FIGURE 3:**
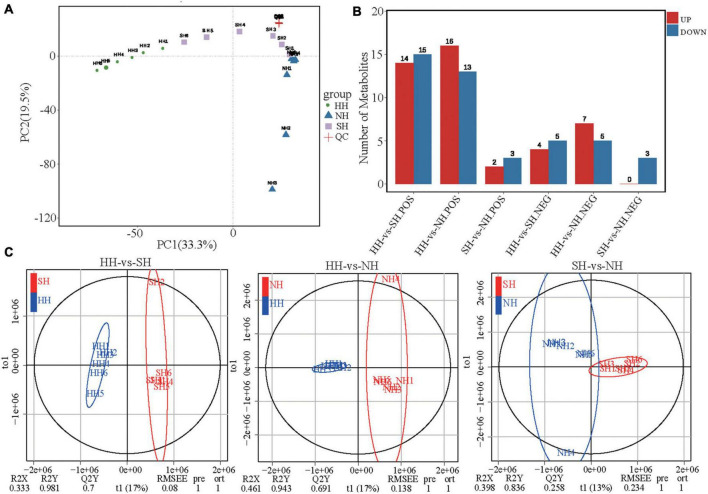
LC-MS/MS analysis longissimus dorsi metabolic profiles for the HH, SH and NH sheep. **(A)** PCA score plots of positive mode; **(B)** Number of up/down-regulated metabolites of different compared groups in POS and NEG modes; **(C)** OPLS-DA of HH-SH, HH-NH and SH-NH comparisons in positive mode.

LC-MS/MS analysis was performed to study the differences in metabolite compositions of the meat diversity of the crossbred sheep populations. PCA analysis results showed that the three groups, including the quality control samples, were independently separated in the principal component (PC)1 × PC2 score plot (Figure 3A and Supplementary Figure 1A). Further analysis *via* OPLS-DA showed clear differences between the meat quality of the three sheep breeds (Figure 3B and Supplementary Figure 1B), thus demonstrating the precision and repeatability of the LC-MS/MS detection. We detected 87 differential metabolites (63 in positive mode and 24 in negative mode, Figure 3C and Supplementary Table 5), which we used for multivariate analysis of the muscle metabolites. Compared with the metabolites in the SH group, 38 differential metabolites were significantly altered in the HH group, of which, 18 were upregulated, and 20 were downregulated. Compared with the NH group, 41 differential metabolites were significantly altered in the HH group, of which, 23 were upregulated, and 18 were downregulated. Compared with the NH group, 8 differential metabolites were significantly altered in the SH group, of which, 2 were upregulated, and 6 were downregulated.

**FIGURE 4:**
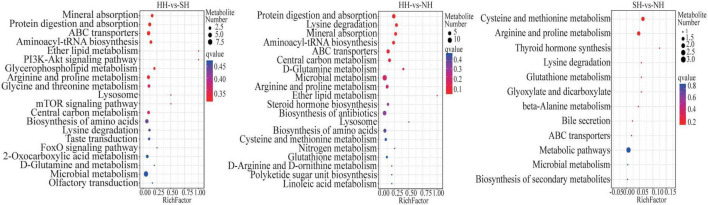
Bubble diagram of top 20 KEGG pathway enrichment in HH-SH, HH-NH, and SH-NH comparisons. The ordinate indicates the pathways.

KEGG enrichment analysis was used to determine the biological mechanisms associated with phenotypic changes. All differential metabolites were co-enriched in 131 biological pathways (*P* < 0.05). In the HH vs SH comparison, 38 differential metabolites were significantly annotated into eight pathways, and most were involved in lipid metabolism-related pathways, such as glycerophospholipid metabolism, protein digestion and absorption, arginine and proline metabolism and ABC transporters (Figure 4). In the HH vs NH comparison, 41 differential metabolites were significantly annotated in 10 pathways (*P* < 0.05), most of which were involved in glycerophospholipid metabolism, protein digestion and absorption, arginine and proline metabolism. In the SH vs NH comparison, two metabolic pathways were significantly enriched in fat metabolism, cysteine and methionine metabolism and arginine and proline metabolism (*P* < 0.05).

### Metabolomic profiling based on GC-MS

**FIGURE 5:**
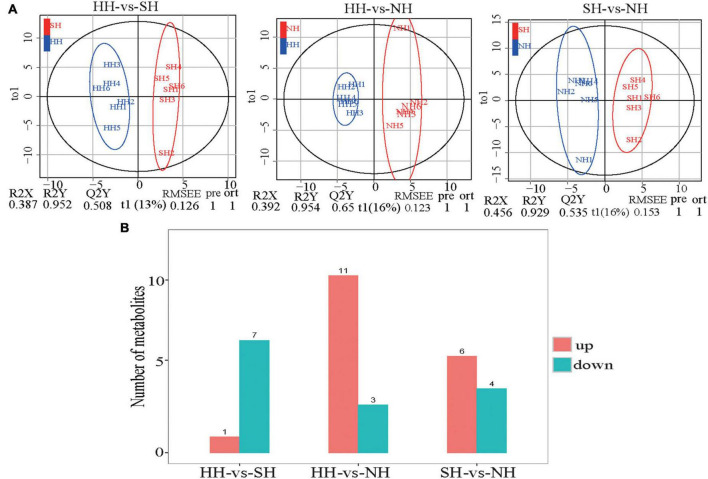
GC-MS/MS analysis longissimus dorsi metabolic profiles for the HH, SH and NH sheep. **(A)** OPLS-DA of HH-SH, HH-NH and SH-NH comparisons; **(B)** Number of up/down-regulated metabolites of different compared groups; red: up-regulated genes; blue: down-regulated genes.

OPLS-DA showed that the meat quality of the three sheep crossbreeds was clearly differentiated (Figure 5A), demonstrating the precision and reproducibility of the non-targeted GC-MS detection results. We detected 32 differential metabolites for further analysis of the muscle metabolites (Figure 5B and Supplementary Table 6). Compared with the metabolites in the SH group, eight differential metabolites were significantly altered in the HH group, of which, one was upregulated, and eight were downregulated. Compared with the NH group, 14 differential metabolites were significantly altered in the HH group, of which, 11 were upregulated, and three were downregulated. Compared with the NH group, 10 differential metabolites were significantly altered in the SH group, of which, six were upregulated, and four were downregulated.

**FIGURE 6:**
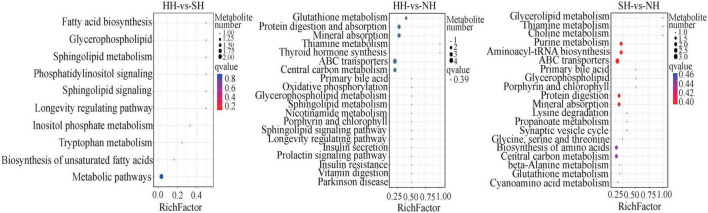
Bubble diagram of top 20 KEGG pathway enrichment in HH-SH, HH-NH, and SH-NH comparisons. The ordinate indicates the pathways.

In total, 80 biological pathways were enriched. In the HH vs SH comparison, 10 differential metabolites were annotated into 10 pathways, most of which were involved in metabolism pathways, such as fatty acid biosynthesis, glycerophospholipid, and tryptophan metabolism (Figure 6). In the HH vs NH comparison, 14 differential metabolites were significantly annotated into 41 pathways, including glycerophospholipid metabolism, protein digestion and absorption, glutathione metabolism and other biological pathways. Similarly, in the SH vs NH comparison, 10 differential metabolites were enriched in 29 metabolic pathways, most of which were related to fat metabolism.

### Integrative analysis of the transcriptome and metabolome

**FIGURE 7:**
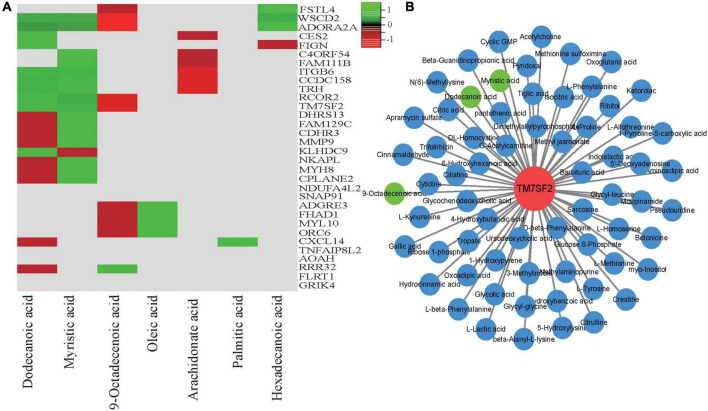
Correlation and pathway analysis of significant differential compounds and verification of the associated DEGs. **(A)** Correlation analysis of the significant differential metabolites and the DEGs; **(B)** Cytoscape representation of candidate gene TM7SF2 and co-expressed differential metabolites involved in lipid metabolism. Hub gene TM7SF2 is in red; related differential metabolites are in blue circles, and the three trait-related metabolites are in green.

To obtain additional biological information *via* transcriptomic and metabonomic analyses of the meat quality diversity, we conducted conjoint biological annotation and correlation testing of the transcriptome and metabolome *via* LC-MS/MS and GC-MS/MS. We performed KEGG pathway annotation (Supplementary Table 7), and the main processes were related to fat-related metabolism pathways, including oxidative phosphorylation, PPAR signaling, and arachidonic acid metabolism, indicating that fatty acid metabolism played an important role in the meat quality of the three hybrid sheep populations. Figure 7A shows several genes and their positive and negative correlations (|r| > 0.5 and *p* < 0.05) with seven meat quality-related fatty acids (dodecanoic, myristic, 9-octadecenoic, oleic, palmitic, arachidonate, and hexadecenoic acid). Among them, *MYH8* and *MYL10* participate in regulating muscle growth and development (Figure 2A) and were correlated with fatty acid levels in meat quality. Additionally, *TM7SF2* was strongly correlated with 66 metabolites, including dodecanoic, myristic, and 9-octadecenoic acid (Figure 7B).

### qRT-PCR validation of functional gene expression

**FIGURE 8:**
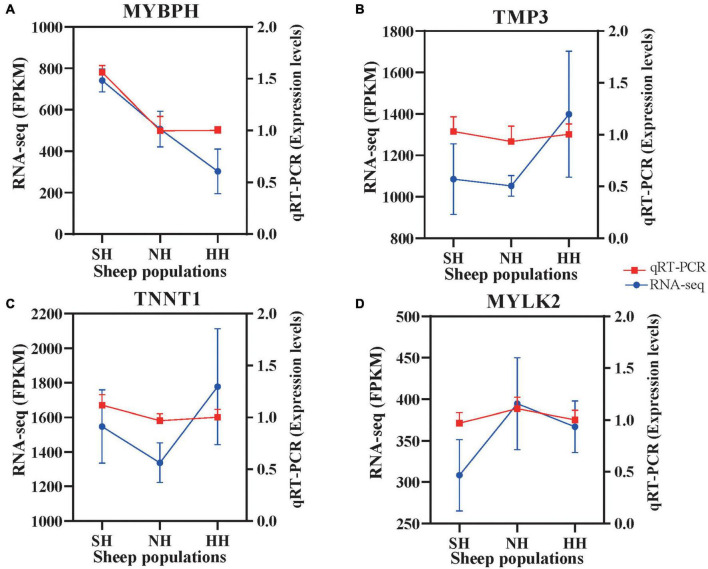
Confirmation of expression patterns of the four selected genes *via* qRT-PCR. The qRT-PCR results were consistent with the RNA-seq data. **(A)** MYBPH; **(B)** TMP3; **(C)** TNNT1; **(D)** MYLK2.

To evaluate the reliability of the RNA-seq, we selected four genes and validated them *via* qRT-PCR. All detected gene mRNA levels were consistent with the RNA-seq results (Figure 8), indicating that the RNA-seq data were reliable.

## Discussion

Hu sheep has many excellent characteristics, including strong fecundity, suitable feeding, rapid early growth, making this breed important in China’s mutton sheep industry. Suffolk and Southdown are one of the most popular breeds for meat production (4, 5), since the introduction of two excellent male parents, the meat production performance has reached a new height after a series of hybridization improvements (4). Previous studies have focused on production performance and meat quality, for example, compared with HH, excellent ram breeds, such as Texel and Suffolk, crossbred with Hu sheep can improve the growth, production, and slaughter performance (21, 22). However, there are few studies on the molecular aspects of different sires how affect meat quality. Here, we performed transcriptomics and metabolomics analyses based on transcriptome sequencing, LC-MS/MS and GC-MS to explore how sires of three hybrid sheep populations affected meat quality of the longissimus dorsi. We focused only on the processes potentially related to growth, development, and meat quality obtained through integrative analyses containing significantly correlated DEGs and differential metabolites. These genes have been reported to be involved in lipid metabolism. Our results may contribute to improving sheep quality at the genetic and molecular levels and to better understanding the molecular mechanisms associated with growth and lipid metabolism.

Sheep growth traits are mainly manifested in muscle growth and development, and the muscle growth rate directly affects meat yield. Muscle development is closely associated with many critical cellular functions and biological processes. Approximately one-third of all muscle proteins are composed of myosin, the most abundant protein in muscles (23). Several myosins are involved in differentiating muscle fibers: type I (slow-twitch, red muscle, oxidative), type IIA (fast-twitch, red muscle, oxidative), and type IIB/IIx (fast-twitch, white muscle, glycolytic). mRNA abundances of MyHC IIb fast-twitch can indicate muscle protein synthesis and muscle growth rates. Some studies suggest that commercial pig breeds have larger proportions of IIb glycolytic fibers and rapid growth rates, which are thought to play important roles in determining meat quality. *MYLK3* is involved in muscle cell development and the Ca^2+^ signaling pathway in striated muscle contraction (24). *MYH8* is a marker of muscle regeneration (25), and studies have shown that *MYH8* expression dominates early skeletal muscle development (26). *MYOM3* is expressed in both mature cardiac and skeletal muscle cells but only in fast-twitch muscle fibers (IIA) (27). In the present study, transcriptomic analysis revealed that *MYLK3*, *MYH8* and *MYOM3* were enriched for GO terms related to growth and development (Figure 2A), including multicellular organismal processes, cellular processes, metabolic processes and catalytic activity, these results were consistent with those of previous studies (28). Compared with the HH sheep, those genes were upregulated in NH and SH sheep (Supplementary Table 3), indicating that these genes may affect the growth of muscles among the NH, SH and HH sheep. These factors may have caused the differences in meat quality and fatty acid profiles among the three hybrid sheep populations.

Sheep meat is an important source of animal protein worldwide and is popular for its tenderness, juiciness, nutritional value and lack of fat. Meat quality is a main complex trait that is comprehensively evaluated through a series of indicators, such as meat color, intramuscular fat content, pH, water-holding capacity, and tenderness (29–32). Additionally, fatty acid metabolism strongly influences meat quality. Previous studies have found that the fatty acid composition and its metabolism in muscles are important factors influencing overall meat quality and consumer preference (15, 33–35). In the present study, enrichment analysis of the muscle metabolomic data revealed several pathways related to fat metabolic processes (Figures 4, 6), including arachidonic acid metabolism and fatty acid biosynthesis. In this analysis, *TM7SF2* expression was significantly higher in the SH sheep than in the HH sheep (Supplementary Table 3). Interestingly, the transcriptomic and metabolomic correlation analysis results showed that *TM7SF2* participates in other metabolic pathways, which is consistent with previous research results (36). We also found that *TM7SF2* and metabolites in the fatty acid metabolism pathway are correlated with dodecanoic (*r* = 0.62), myristic (*r* = 0.71) and 9-octadecenoic acid levels (*r* = 0.67) (Figures 7A,B). *TM7SF2* is considered a crucial gene that regulates *SREBP* (Sterol Regulatory Element Binding Protein 2) and is involved in lipid and lipoprotein metabolism (36, 37). Therefore, the high *TM7SF2* expression levels in the SH sheep compared with those of the HH sheep and the high abundances of dodecanoic, myristic and 9-octadecenoic acid components suggest that *TM7SF2* is responsible for the larger accumulation of fatty acid levels and affects the meat quality according to the gene-metabolite network.

Fatty acids are important chemical substances constituting fats and are important aromatic substances or precursors of aromatic substances. Studies have shown that fatty acid metabolism levels and composition affect meat quality and its nutritional value; for example, dodecanoic (C12:0), myristic (C14:0), palmitic (C16:0), stearic (C18:0), linoleic (C18:1), alpha-linolenic (C18:2) and linolenic fatty acids (C18:3) can enhance meat flavors (38–41). Dodecanoic acid is reported to enhance intramuscular fat deposition and increase the accumulation of myristic and myristoleic acids (C14:1), which may benefit human health (42). Myristic acid can increase high-density lipoprotein (“good”) cholesterol more than can any other fatty acid (43). Increased palmitic acid can improve meat stability and quality (44). Additionally, studies have shown that oleic acid is important in the meat quality of Japanese Black cattle (45). Here, we found significantly differences in the metabolism levels of some acids among HH, NH and SH sheep (Supplementary Tables 5, 6). Dodecanoic and myristic acid levels were higher in NH and SH sheep than in HH sheep, and myristic, oleic and palmitic acid levels were higher in NH sheep than in SH sheep. Previous studies have shown that hybridization can improve the quality of meat products (1, 2). These results suggest that sires of different hybrid sheep breeds cause differences in muscular fatty acid contents, ultimately leading to differences in meat quality.

## Conclusion

In this study, a great number of DEGs and differential metabolites were identified based on the integrative analysis of transcriptomics and metabolomics in hybrid sheep’ muscle. Different hybrid male parents caused differential expression of meat quality-related genes, metabolites, and related pathways, in different hybrid populations, such as fatty acid metabolism (particularly arachidonic acid metabolism, fatty acid biosynthesis and lipolysis in adipocytes) and lipid metabolism-related pathway. But most importantly, fatty acid metabolism played a key role in the meat quality of the three hybrid sheep populations. Overall, these results will provide effective information and more evidence to support further insight into the key genes and metabolites involved in muscle growth and meat quality in hybrid sheep populations.

## Data availability statement

The data presented in this study are deposited in the SRA repository, accession number: PRJNA859945, and our data have been released.

## Ethics statement

The studies involving animals were reviewed and approved by the Institutional Animal Care and Use Committee of Lanzhou Institute of Husbandry and Pharmaceutical Science of Chinese Academy of Agricultural Sciences (Ethic approval file No. NKMYD201805).

## Author contributions

ZL, BC, and BY conceived and designed the study. YY, JBL, DZ, and JYL collected the samples. BC, CY, and TG performed the experiments and analyzed the data. BC wrote the manuscript. BC and ZL contributed to revisions of the manuscript. All authors read and approved the manuscript.

## Footnotes

1
https://cran.r-project.org/web/packages/Hmisc/index.html

2
https://cytoscape.org/

3
https://www.ncbi.nlm.nih.gov/assembly/GCF_000298735.2/

## Supplementary material

Supplementary Figure 1 LC-MS/MS analysis longissimus dorsi metabolic profiles of negative mode for the HH, SH and NH sheep. **(A)** PCA score plots; **(B)** OPLS-DA of HH-SH, HH-NH and SH-NHcomparisons; **(C)** Cluster heatmap of different metabolites of HH-SH, HH-NH and SH-NH comparisons, red: upregulated genes; blue: downregulated genes.

Supplementary Table 1 Primers of genes selected for qRT-PCR.

Supplementary Table 2 Summary of the sequencing reads alignment to the reference genome.

Supplementary Table 3 DEGs of HH-SH, HH-NH and SH-NH comparisons.

Supplementary Table 4 GO analysis of the DEGs in the HH-SH, HH-NH and SH-NH comparisons.

Supplementary Table 5 Differential metabolites of HH-SH, HH-NH and SH-NH comparisons based on LC-MS/MS.

Supplementary Table 6 Differential metabolites of HH-SH, HH-NH and SH-NH comparisons based on GC-MS.

Supplementary Table 7 KEGG pathway enrichment of HH-SH, HH-NH, and SH-NH comparisons based on integrative analysis.
