# Supplementary figures and images for: Transcriptome-metabolome analysis reveals how sires affect meat quality in hybrid sheep populations
Source: Front Nutr. 2022 Aug 11;9:967985. doi: 10.3389/fnut.2022.967985 (PMC9403842; doi:10.3389/fnut.2022.967985)

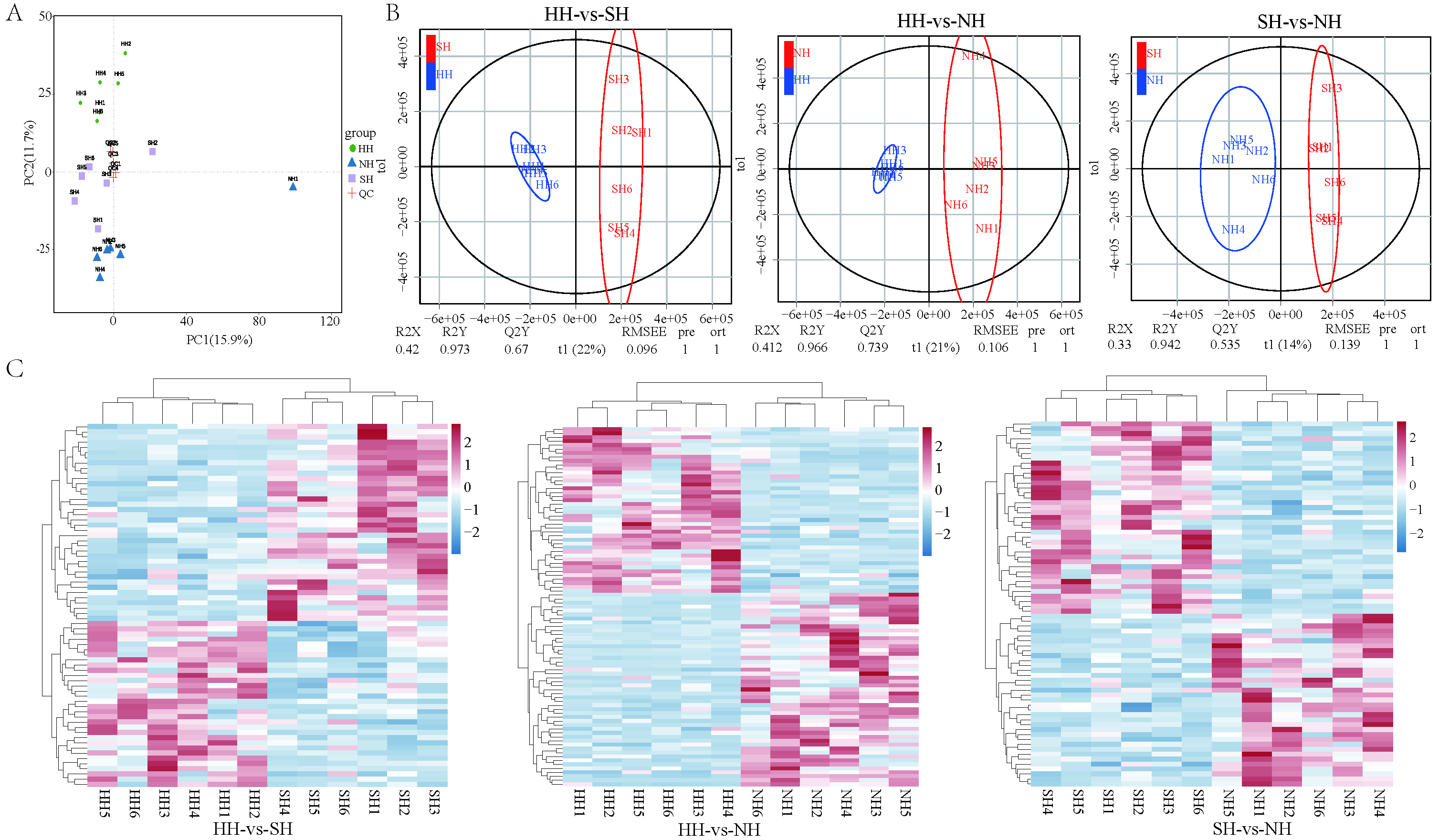

Supplement: Supplementary Figure 1 — LC-MS/MS analysis longissimus dorsi metabolic profiles of negative mode for the HH, SH and NH sheep. (A) PCA score plots; (B) OPLS-DA of HH-SH, HH-NH and SH-NHcomparisons; (C) Cluster heatmap of different metabolites of HH-SH, HH-NH and SH-NH comparisons, red: upregulated genes; blue: downregulated genes. [file Image_1.TIFF]
