# Supplementary material for: Transcriptome-metabolome analysis reveals how sires affect meat quality in hybrid sheep populations
Source: Front Nutr. 2022 Aug 11;9:967985. doi: 10.3389/fnut.2022.967985 (PMC9403842; doi:10.3389/fnut.2022.967985)
Supplement: Supplementary Table 1 — Primers of genes selected for qRT-PCR. [file Table_1.docx]

Table S1. Primers of genes selected for qRT-PCR

| Gene | Accession No. | Primer sequences (5'-3') | Products length (bp) |
| --- | --- | --- | --- |
| MYBPH | XM_027976179.2 | F: GAGGCCAAAGCAGCCATCAACA  R: GCGCTCGATCACCGTGAACC | 198 |
| MYLK2 | XM_042229986.1 | F: CCTGGCACGGAGGTATAACCC  R: CCCCAGGCTCCACATGTCT | 124 |
| TNNT1 | KT218691.1 | F: ACGCTCATCGACGTCCACT  R: CTCGCTCCTTCTCGGTTCTGA | 130 |
| TMP3 | XM_027975562.2 | F: TTGCCGAGAGATGGATGAGCAAA  R: GGGTCTCTGCCTCCTTGAGT | 146 |
| ACTB | NM_001009784.3 | F: CCAACCGTGAGAAGATGACC  R: CCCGAGGCGTACAGGGACAG | 97 |
