# Supplementary material for: Transcriptome-metabolome analysis reveals how sires affect meat quality in hybrid sheep populations
Source: Front Nutr. 2022 Aug 11;9:967985. doi: 10.3389/fnut.2022.967985 (PMC9403842; doi:10.3389/fnut.2022.967985)
Supplement: Supplementary Table 2 — Summary of the sequencing reads alignment to the reference genome. [file Table_2.DOCX]

Table S2. Summary of the sequencing reads alignment to the reference genome.

| Sample | Clean reads | Total reads | Unmapped | Unique Mapped | Multiple Mapped | Total Mapped |
| --- | --- | --- | --- | --- | --- | --- |
| HH1 | 45,111,858 | 44,989,514 | 7.39% | 77.44% | 15.17% | 92.61% |
| HH2 | 46,145,670 | 45,963,718 | 7.43% | 78.32% | 14.26% | 92.57% |
| HH3 | 42,046,394 | 41,933,534 | 7.78% | 79.64% | 12.58% | 92.22% |
| HH4 | 50,406,702 | 50,285,760 | 7.32% | 79.90% | 12.78% | 92.68% |
| HH5 | 49,198,502 | 49,122,806 | 8.32% | 78.86% | 12.82% | 91.68% |
| HH6 | 52,405,016 | 52,323,272 | 7.75% | 81.22% | 11.03% | 92.25% |
| NH1 | 53,169,528 | 52,998,724 | 7.73% | 81.58% | 10.69% | 92.27% |
| NH2 | 44,669,044 | 44,602,324 | 7.96% | 82.35% | 9.69% | 92.04% |
| NH3 | 44,242,634 | 44,185,306 | 7.60% | 80.94% | 11.46% | 92.40% |
| NH4 | 39,445,416 | 39,408,782 | 7.90% | 82.40% | 9.70% | 92.10% |
| NH5 | 40,337,148 | 40,282,036 | 7.50% | 79.79% | 12.71% | 92.50% |
| NH6 | 39,964,748 | 39,922,524 | 8.01% | 81.21% | 10.78% | 91.99% |
| SH1 | 52,360,950 | 52,315,848 | 8.53% | 82.45% | 9.03% | 91.47% |
| SH2 | 41,169,946 | 41,088,292 | 6.86% | 81.36% | 11.77% | 93.14% |
| SH3 | 48,411,890 | 48,298,394 | 7.66% | 80.51% | 11.84% | 92.34% |
| SH4 | 46,723,740 | 46,680,238 | 8.30% | 80.74% | 10.96% | 91.70% |
| SH5 | 42,322,836 | 42,248,866 | 7.88% | 80.44% | 11.69% | 92.12% |
| SH6 | 43,630,384 | 43,574,152 | 7.84% | 81.90% | 10.26% | 92.16% |
